# Influence of Water Treatment and Wastewater Treatment on the Changes in Residues of Important Elements in Drinking Water

**DOI:** 10.3390/molecules27030972

**Published:** 2022-02-01

**Authors:** Jacek Cybulski, Agata Witczak, Kamila Pokorska-Niewiada

**Affiliations:** Department of Toxicology, Dairy Technology and Food Storage, Faculty of Food Sciences and Fisheries, West Pomeranian University of Technology in Szczecin, Papieża Pawła VI Street 3, 71-459 Szczecin, Poland; agata.witczak@zut.edu.pl (A.W.); kamila.pokorska@zut.edu.pl (K.P.-N.)

**Keywords:** drinking water, toxic elements, trace elements, treatment of water and wastewater

## Abstract

Drinking water is the essential medium for food production, and is also needed for direct consumption; while it must be free of harmful substances, it also must have a composition that is beneficial to health. The aim of this study was to evaluate the influence of water and wastewater treatment on reducing the concentrations of zinc (Zn), nickel (Ni), iron (Fe), manganese (Mn), copper (Cu), lead (Pb), and arsenic (As) in the Western Pomerania Voivodeship in Poland. The research was carried out in 2017–2019. The analysis was performed with inductively coupled plasma atomic emission spectrophotometry (ICP-AES). The concentrations of trace elements in drinking water were below maximum acceptable concentrations (MACs). Reductions in the most dangerous elements during water treatment fluctuated from 48.5% (As) to 97% (Pb). Wastewater treatment reduced the concentrations of analyzed elements by a range of 28.6 to 60.8%, and the most toxic elements (Pb and As) by over 50%. Trace element concentrations in treated wastewater were below MAC values, and ranged from 1.15% (Pb) to 6.23% (As) of MACs for toxic elements. The concentrations of both essential elements (Zn, Ni, Fe, Mn, Cu) and toxic elements (Pb, As) in drinking water were below the MACs. Water treatment had a significant (*p* < 0.05) effect on decreasing trace element concentrations.

## 1. Introduction

Water distributed to households and food industry plants through water supply networks must meet the highest quality standards, which is why it is subjected to water treatment. The effectiveness of treatment affects the lives of consumers, which is why as technology develops at water treatment and wastewater treatment facilities, various techniques are combined with the aim of increasing treatment efficiency [1]. Drinking water can be a valuable source of many essential elements for humans, such as zinc, iron, manganese, and copper. 

Trace elements are components of the Earth’s crust, released into the waters naturally, but the cause of observed exceedance values is mainly the result of anthropogenic activity [2]. 

Drinking water, in the context of the human body’s high demand for its consumption, can be an important source of harmful elements to organisms [3]. For example, arsenic causes various forms of cancer, and its accumulation in the thyroid gland causes endemic goiter. Another extremely dangerous element is lead, which damages the central and peripheral nervous systems, intestines, and kidneys; it also blocks the action of many enzymes, and prevents the formation of vitamins [4].

The increasing use of trace elements in industry can also lead to increased concentrations of them in wastewater treatment plants [1]. Unfortunately, thus far, little research has focused on trace element concentrations in wastewater and their behavior in wastewater treatment, although they may have potential value if recovered from wastewater treatment plant sludge [2]. Therefore, it is extremely important to have quantitative information on the concentrations of trace elements in wastewater that returns to surface water after treatment and, potentially, to drinking water. In the European Union (EU) countries, the quality of water intended for human consumption is regulated by Directive 2020/2184 of 16 December 2020, and in Poland, additionally, by the Regulation of the Minister of Health of 2017 [5,6]. This directive includes, inter alia, information on the permissible content of selected elements, such as copper (2 mg L^−1^), nickel (20 μg L^−1^), iron (200 μg L^−1^), manganese (50 μg L^−1^), lead (5 μg L^−1^), and arsenic (10 µg L^−1^).

The impulse to assess the impact of water and wastewater treatment processes on the reduction in zinc, nickel, iron, manganese, copper, lead, and arsenic concentrations was prompted by the high ability of the human body to bioaccumulate these trace elements, including potentially toxic ones. The second reason was the recommended consumption of drinking water of at least 2.0–2.5 L per day [7].

The article is part of a project that analyzes the impact of water treatment and wastewater treatment on the quality of drinking water in terms of many parameters, including the reduction in the concentrations of organic compounds, such as organochlorine pesticides [8], and inorganic compounds, including trace elements. The study was carried out in the Voivodeship of Western Pomerania in Poland. The authors draw attention to the often overlooked aspects of water and wastewater treatment efficiency in different seasons of the year. It is extremely important to fit the water and wastewater treatment accordingly to the season of the year, as different biochemical processes occur in the waters at different times of the year, and this can affect the efficiency of treatment.

## 2. Results

### 2.1. Analyzed Elements in Drinking Water and Wastewater

Table 1 presents the mean contents of the elements analyzed in raw and treated drinking water and in raw and treated wastewater.

The contents of zinc in raw and treated water fluctuated within a range of 0.008–0.069 mg L^−1^ (detected in 100% of samples) and < LOD–0.021 mg L^−1^ (in 98% of samples), respectively. In raw and treated wastewater, the Zn content determined was 0.088–0.336 mg L^−1^ (100% of samples) and 0.035–0.192 mg L^−1^ (100% of samples), respectively (Table 1). The analysis of the dependence between the water parameters and the zinc quantities determined did not indicate significant correlations in drinking water, but in raw drinking water in summer and autumn was positively correlated with ammonium ion content (r = 0.836, r = 0.576, respectively) (Table 2). In autumn, a positive correlation with chemical oxygen demand (COD) (r = 0.576) was also observed. In wastewater, however, a significant correlation was only confirmed in treated wastewater. Therefore, in spring, positive dependencies of zinc were confirmed with biochemical oxygen demand (BOD_5_) (r = 0.720) and negative ones with nitrogen (r = −0.738), in summer with COD (r = 0.918), and in autumn with COD (r = −0.738) and phosphorus content (r = −0.721). When comparing the zinc concentrations in different seasons of the year (Table 2), it was noted that in drinking water significant differences (*p* < 0.05) occurred between the water abstracted in summer and winter; a similar dependency was noted in raw wastewater.

The nickel content in raw and treated water fluctuated within the ranges of < LOD–0.004 mg L^−1^ (detected in 98% of the samples) and < LOD–0.003 mg L^−1^ (in 52% of the samples), respectively. The Ni contents confirmed in raw and treated wastewater were 0.010–0.068 mg L^−1^ (100% of samples) and 0.006–0.041 mg L^−1^ (100% of samples), respectively (Table 1). Throughout the two-year study period, no significant dependencies between the biochemical parameters of drinking water and raw drinking water and the quantities of nickel determined were confirmed. Only in treated wastewater in autumn and winter was the quantity of COD positively correlated with nickel content (r = 0.639 and r = 0.756, respectively). The content of total nitrogen in autumn, however, was positively correlated with nickel (r = 0.744), while in winter it was negatively correlated (r = −0.693) (Table 2). The quantity of nickel in the samples tested differed significantly (*p* < 0.05) between the seasons of the year—in drinking water, between spring and autumn; in raw drinking water, between summer and winter; and in raw wastewater, between summer and autumn (Table 3). Positive dependencies were noted in comparisons between nickel and other elements in raw drinking water between the seasons of the year—in spring with the level of arsenic (r= 0.695), in summer with the level of copper (r = 0.633), and in autumn with the level of lead (r = 0.641). In raw wastewater, in autumn, a negative correlation was observed between nickel and the content of copper (r = −0.666), while a positive correlation was noted with the content of arsenic (r = 0.815) (Table 4). The content of iron in raw and treated water fluctuated within ranges of 0.002–0.068 mg L^−1^ (detected in 100% of samples) and 0.001–0.010 mg L^−1^ (in 100% of the samples), respectively. In raw and treated wastewater, the contents of Fe confirmed were 0.058–1.863 mg L^−1^ (100% of the samples) and 0.028–0.377 mg L^−1^ (100% of the samples), respectively (Table 1). In spring and autumn, biochemical parameters of water and wastewater—such as COD, BOD_5_, total phosphorus, and total nitrogen—did not affect iron levels (Table 2). In summer the quantity of chlorine dioxide affected (r = 0.749) the level of iron in drinking water. A negative correlation of iron with the content of nitrates (V) (r = −0.622) was observed in the raw drinking water sampled in summer. In winter, a negative correlation was noted between BOD_5_ content and iron in both treated and raw wastewater; furthermore, a positive correlation with COD was confirmed in treated wastewater in winter (Table 2). In summer, negative dependencies were observed between the contents of Fe and phosphorus and the content of total nitrogen in raw wastewater. Significant differences (*p* < 0.05) were noted in the iron content of treated wastewater between spring and autumn. In the other seasons, no changes were observed (Table 3). In raw drinking water and treated wastewater collected in spring, a significant (*p* < 0.05) positive correlation was noted between iron and arsenic, while when collected in autumn a positive correlation was observed for zinc (Table 4). Furthermore, the analysis of the wastewater samples collected in winter indicated a positive correlation with lead.

The content of manganese in the water analyzed did not exceed 0.02 mg L^−1^, and in wastewater it did not exceed 0.240 mg L^−1^ (Table 1). In spring, the biochemical parameters of the water and wastewater did not affect the levels of this element, while in summer, only in raw wastewater was a negative correlation observed in the contents of total phosphorus (r = −0.650) and nitrogen (r = −0.637) (Table 2). In autumn, significant differences were noted depending on the biochemical parameters of the raw drinking water and treated wastewater, while in winter positive correlations were noted between Mn and the contents of nitrates (V) (r = −0.609) and nitrates (III) (r = 0.820). Furthermore, in drinking water, a negative dependency was observed with the content of chlorine dioxide (r = −0.626) (Table 2). When considering the dependencies between elements, only one positive correlation was noted, with the content of copper in treated wastewater collected in spring (Table 4).

The copper content in raw and treated water fluctuated within the ranges of < LOD–0.008 mg L^−1^ (detected in 98% of the samples) and < LOD–0.005 mg L^−1^ (in 69% of the samples), respectively. The content of Cu noted in raw and treated wastewater was 0.011–0.090 mg L^−1^ (100% of samples) and 0.008–0.040 mg L^−1^ (100% of samples), respectively (Table 1). Biochemical parameters slightly affected the quantity of copper in the samples analyzed. The only changes were noted in drinking water and raw drinking water (Table 2). Conversely, significantly (*p* < 0.05) higher copper content was noted in treated wastewater in autumn than in the other seasons of the year. Raw wastewater was characterized by the lowest level of this element in winter, and these differences were largely statistically significant (Table 3). Correlations between Cu and As content were noted of raw wastewater in spring and autumn—in spring it was a positive correlation (r = 0.781), while in autumn it was negative (r = −0.606) (Table 4).

The content of lead in raw and treated water fluctuated within ranges of 0.0005–0.006 mg L^−1^ (detected in 100% of samples) and < LOD–0.001 mg L^−1^ (in 8% of samples), respectively. The contents in raw and treated wastewater were < LOD–0.056 mg L^−1^ (98% of the samples) and 0.003–0.011 mg L^−1^ (100% of the samples), respectively (Table 1). 

The content of arsenic in raw and treated water fluctuated within ranges of 0.0001–0.004 mg L^−1^ (detected in 100% of samples) and < LOD–0.002 mg L^−1^ (in 77% of samples), respectively. The As content in raw and treated wastewater was 0.007–0.022 mg L^−1^ (100% of the samples) and 0.003–0.018 mg L^−1^ (100% of the samples), respectively (Table 1). Changes in the biochemical parameters had a limited effect on lead and arsenic contents, except in raw drinking water in spring and summer and in wastewater in winter (Table 2). The content of lead in raw drinking water differed significantly between summer and autumn. A significant difference in lead concentration was also observed in raw wastewater between spring and autumn (Table 3). The lowest arsenic content was noted in raw wastewater in spring, which was statistically significant in comparison to that in summer and winter. In turn, the highest content of this element was observed in treated wastewater in summer in comparison to the other seasons of the year. 

### 2.2. Analyzed Elements in Drinking Water and Wastewater

**Figure 1:**
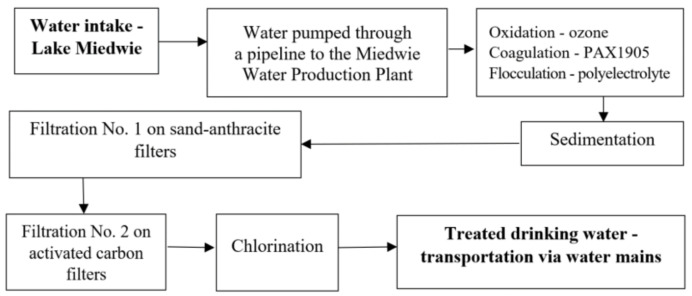
Diagram of the water treatment process at the Żelewo water production plant.

**Figure 2:**
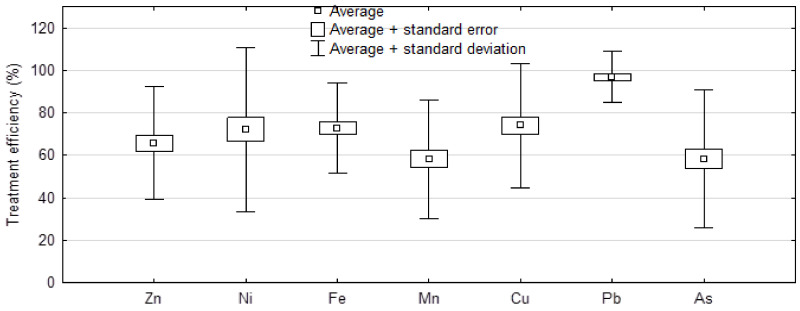
Mean treatment efficiency (%) of water.

**Figure 3:**
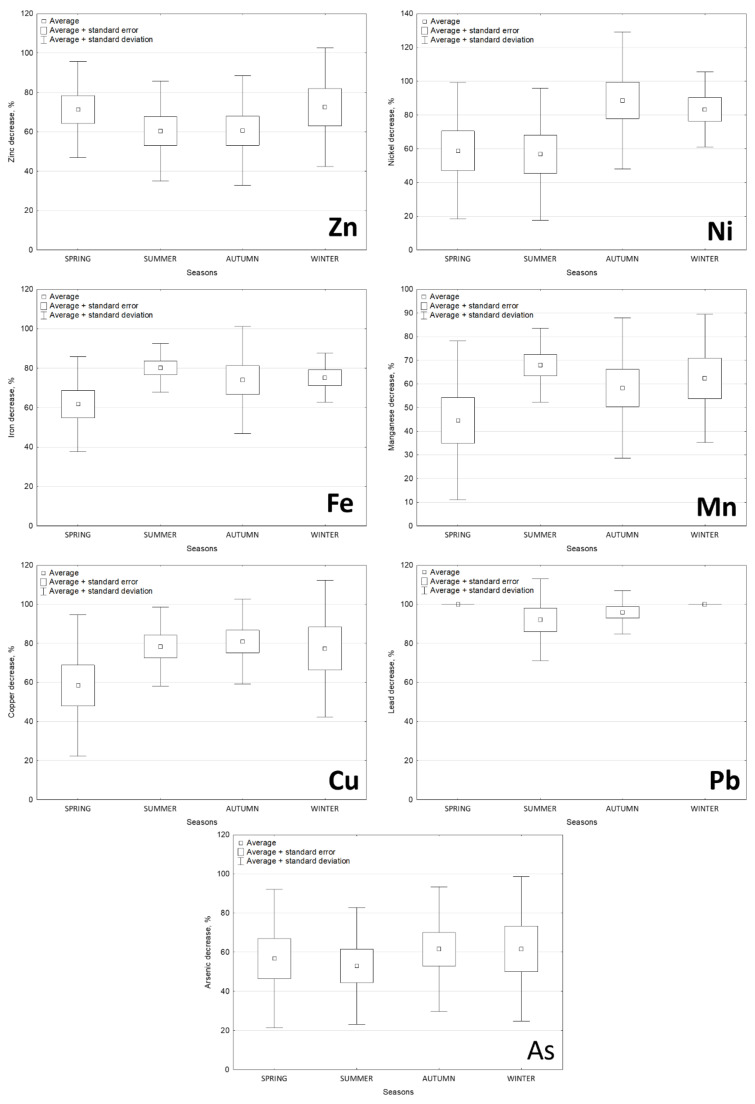
Treatment efficiency (%) of water in different seasons.

The analysis of the effects of water treatment on changes in the content of selected elements (Figure 1) confirmed a significant (*p* < 0.05) decrease in trace elements (in a range of 48.5 to 97%) (Figure 2 and Figure 3, Table 1). This is evidence of the high efficiency of the water treatment methods applied. The lowest treatment efficiency was noted with regard to As and Mn, the contents of which decreased after treatment by 48.5 and 57%, respectively. The highest treatment efficiency was confirmed for Pb (97%) (Figure 2 and Figure 3). 

### 2.3. Estimating Health Risks Associated with Drinking Water Consumption

Estimations of the health risks associated with drinking water consumption were based on comparing ingested amounts with the provisional tolerable weekly intake (PTWI) for each of the trace elements. PTWI values for each of the elements are presented in Table 5. At a presumed mean daily water consumption of 2 L [7] and an adult body weight of 70 kg, none of the mean weekly intakes of the selected elements was exceeded. The minimum and maximum the minimum and maximum ranges of PTWI values for the elements was from 0.003% for iron to 2.66% for arsenic (Table 5).

### 2.4. Effectiveness of Treating Wastewaters in Different Seasons of the Year

**Figure 4:**
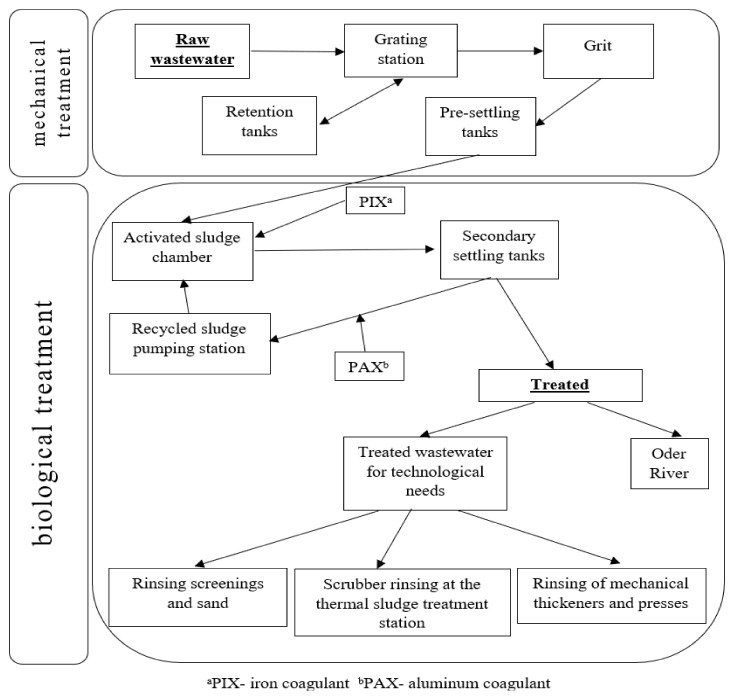
Diagram of mechanical and biological treatment at the Pomorzany wastewater treatment plant.

**Figure 5:**
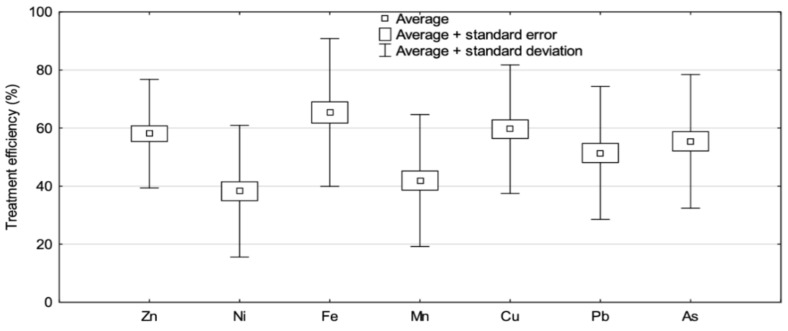
Mean treatment efficiency (%) of wastewater.

**Figure 6:**
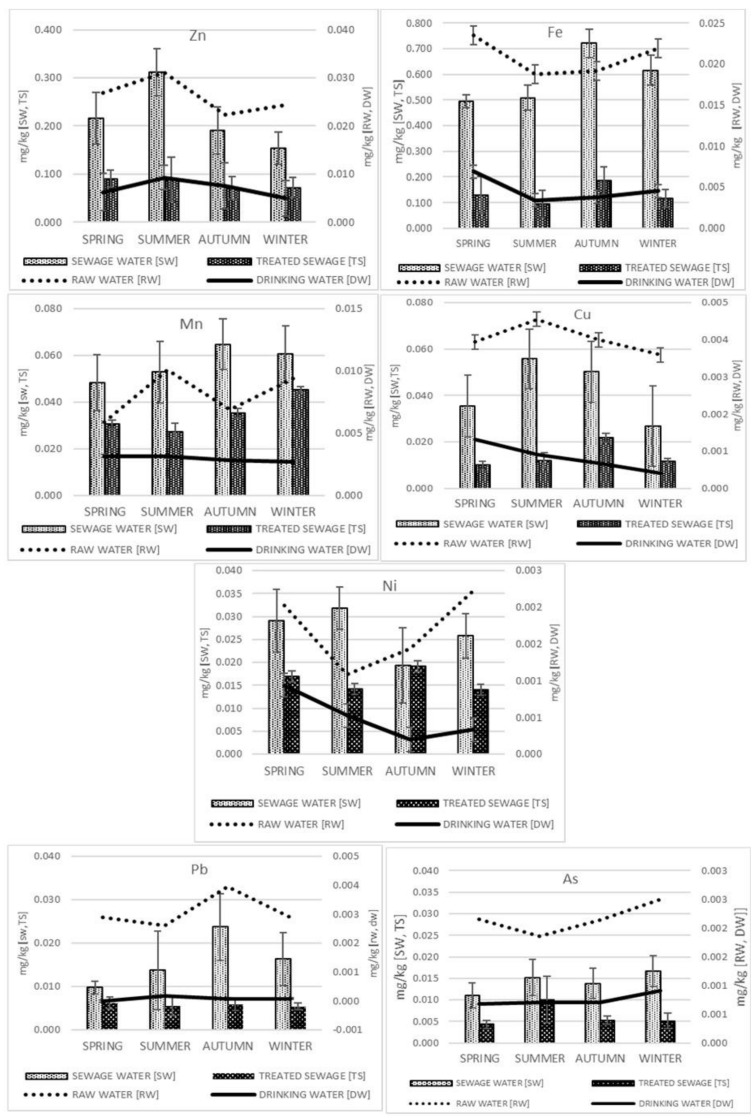
Treatment efficiency (%) of wastewater in different seasons.

The quality of drinking water in urban agglomerations is affected significantly by how municipal wastewaters are drained and treated since, after treatment, they are discharged directly into surface water bodies (treated wastewater from the Szczecin agglomeration is discharged into the Oder River). The analysis of the effects of wastewater treatment (Figure 4) confirmed significant (*p* < 0.05) decreases in trace element residues (in a range of 28.6–60.8%) (Figure 4 and Figure 5, Table 1); this is evidence that the methods applied to treat wastewater are adequately effective. Treatment was least effective for Ni and Mn, the decreases in which after treatment were 28.6 and 34.8%, respectively (Figure 6). The highest treatment effectiveness was noted for Fe (60.8%). Considering that treated waters are discharged into water bodies, they could be an additional source of trace elements for aquatic organisms and their consumers, which indirectly include consumers of drinking water. The concentrations of trace elements in all of the treated wastewater samples from the Pomorzany plant were below the maximum acceptable concentration (MAC) values, and the ranges were from 1.15% (Pb) to 6.23% (As) of the MAC values [10]. 

## 3. Discussion

Concentrations of trace elements in water result from both natural and anthropogenic conditions. Natural conditions include physicochemical properties such as water solubility, pH, redox potential, and the capability of forming soluble complexes [11]. Anthropogenic conditions include advances in civilization and industrial development, inter alia, in the mining and tanning industries, metallurgy, the fertilizer industry, pesticide production, ore refineries, and the pulp and paper industry, all of which, by producing wastewater rich in trace elements, significantly contribute to the accumulation of these compounds in aquatic environments, and they are often difficult to remove with routine treatment methods [12]. Some trace elements are essential microelements for the human body, such as copper, manganese, iron, and zinc [13]. The nutrient reference values (NRVs) for these elements indicate the quantities in which they should be found in the diet via foods and fluids consumed (Table 6). A substantial group, however, comprises toxic elements that have the tendency to bioaccumulate [14]. Organizations such as the WHO, the United States Environmental Protection Agency (US EPA), and the EU all strive to effectively reduce the emission of trace elements into the environment by formulating strict regulations on the quality of wastewater discharged by industry. Unfortunately, values differ between the organizations, rendering it difficult to effectively mitigate the devastation of the natural environment. For example, the WHO set the maximum permissible concentrations of Cu and Pb in mining and galvanizing wastewaters at 2.0 and 0.01 mg L^−1^, respectively [14], while the US EPA levels are 1.3 and 0.015 mg L^−1^, respectively [15]. 

In Poland, regulations in force that govern the MAC values for trace elements in surface waters, drinking water, and treated wastewater discharged into waters are found in the following issues of the Polish Journal of Laws: item 1747 (2019), item 2294 (2017), and item 1311 (2019), respectively [6,10,17]. No values exceeding the allowable limits were noted in any of the materials tested (Table 1). These results indicated that the average adult ingested barely 0.03 to 0.16 NRV in drinking water, while water treatment decreased the NRVs ingested by 27–36.4%. In the study presented in this paper, the water abstracted from Lake Miedwie and subjected to multistage treatment was characterized by safe quantities of all of the elements analyzed that were below the MACs [17] (Table 7). The tests on drinking water performed for the present study, similarly to those carried out in 2005–2006 in Poland on metal concentrations, indicated that these elements’ concentrations were below approximately 10% of the maximum acceptable concentrations [18]. Only the iron content in the water from 16 years ago slightly exceeded the MAC, which could have stemmed from the older water and wastewater infrastructure in operation in Szczecin at the time. Many correlations between elements were noted in the present study; however, no information regarding this was found in the available literature. Only Rahman (2021) reported similar findings in the strong positive correlation of As with Fe in water in southwestern Bangladesh [19]. 

The concentrations of elements (As, Pb, Ni, Mn, Fe, Cu, Zn) confirmed in raw drinking water were below the MACs (Table 1 and Table 7). Compared to the studies of many other authors, the quality of water in Lake Miedwie was characterized by high quality parameters. However, despite the modern solutions applied at the water and wastewater treatment plants, unnecessary trace elements were not completely removed. Water treatment reduced the concentrations of specific trace elements within a wide range from 48.5 (As, Mn) to 97% (Pb); however, wastewater treatment was less effective, as it reduced the concentrations of trace elements by 28.6 (Ni, Mn) to 60.8% (Fe). Drinking water from Lake Miedwie did not exceed the standards in any of the study periods. A comparative analysis of drinking water from various European countries indicated that MACs (4.63%) were exceeded [12,20]. Elevated trace element contents in water and wastewater were confirmed in the period from spring to early autumn. The higher trace element contents in raw drinking water and drinking water during this period could have been linked with the higher flow of elements from sediments to the water that occurs as temperatures increase [21]. Presumably, the use of coagulants in the treatment plants significantly affected the reduction in the concentrations of trace elements. PAX-1905, a high-basicity coagulant, was used at the Żelewo water production plant. Zinc occurring in water in dissolved form is a component of enzymes, and is a catalyst in many reactions. The content of this element in water is highly variable, and depends on geological formations and pollutants from many sectors, including pigment production, battery construction, and ammunition manufacture [14]. The zinc content in potable groundwater from different parts of the world fluctuates within a range of 15–80 µg L^−1^. It was determined that in the 2017–2019 period it was 0.007 ± 0.004 mg L^−1^. The US EPA determined that the permissible zinc content in treated wastewater was 2 mg L^−1^ [15], which is fourfold higher than in treated wastewater tested in the present study (0.54 mg L^−1^). 

The effectiveness of filtration through filtration beds greatly affects the content of trace elements in treated water. While trace elements are, in fact, retained in beds, they are not permanently bound to them. Filtration bed contamination is one of the reasons that there are trace elements in drinking water [21]. Nickel occurs in water primarily as [Ni(H_2_O)_6_]^2+^ ions, and is part of the active sites of many enzymes [22]. Because of the potentially high toxicity of this element, its recommended dietary allowance (RDA) has not been determined. Nevertheless, many studies confirm that the estimated daily consumption of nickel in food and water globally is 80–130 µg per day [23]. Water collected from the surface waters of Woji Creek, Rivers State, Nigeria in 2019 was confirmed to have a mean Ni concentration of 0.3545 ± 0.1652 mg L^−1^ [24]; this value was significantly higher than those obtained in the present study. 

Manganese and iron are among the most common trace elements in aquatic environments. A large percentage of the population of the Baltic states is at risk of potential exposure to elevated levels of manganese and iron in drinking water, since approximately 30% of groundwater samples collected exceeded the standards for these elements set forth in the European Union Council Directive 2020/2184 on the quality of water intended for human consumption [5]. Although these are essential nutrients, when they occur in high concentrations in drinking water they are linked with various health problems. Iron that occurs in water as Fe^+2^ and Fe^+3^ ions is responsible for tissue respiration [14]. 

As is the case with other elements, the acceptable iron concentration in drinking water varies in many guidelines. For example, Turkish drinking water standards [25] permit 200 μg L^−1^ of iron, while the US EPA (2006) limit is 300 μg L^−1^ [15]. The limit for manganese is 50 μg L^−1^, which is the same as that in the TDWS (2005) and the US EPA guidelines (2006) [15,25]. Manganese plays defensive roles in cells, provides protection against reactive oxygen species, and also regulates the urea cycle and proper dopamine production [14]. Tap water sampled from Eskisehir Province in the Central Anatolian Region of Turkey in 2013 had an iron level of 110 µg L^−1^ and a manganese level of 104 µg L^−1^ [26]. These levels were extremely high in comparison to the drinking water analyzed in the present study, in which iron and manganese did not exceed 10 µg L^−1^ and 7 µg L^−1^, respectively. Copper occurs in water as Cu^+^ ions, and it participates, inter alia, in the formation of crosslinks in collagen, elastin, and melanin, as well as in maintaining keratin structure. The toxicity of copper in the aquatic environment depends primarily on the alkalinity of the water, and also on its hardness. Copper is less toxic in more alkaline, harder water, as it is less available due to the formation of copper carbonate complexes; this is why the toxicity of copper increases with decreasing water alkalinity and hardness, pH, dissolved oxygen concentration, chelating agents, humic acid content, and suspended matter content [27]. 

The occurrence of lead in drinking water is undesirable, as it provides no known health benefits, while the negative effects from it are many—the most important of which is lead poisoning. Lead can affect nearly all of the organs and systems in the human body, and it can cause serious damage to the brain, kidneys, nervous system, and reproductive system [24]. Due to its low alkalinity and buffering capacity, soft water is more dangerous because of the greater mobility of lead in the form of soluble salts [12], while water that is hard and highly alkaline (and also with higher pH values) contains sparing or practically insoluble lead salts, such as lead phosphate, lead sulfate, lead hydroxide, lead carbonate, and basic lead carbonate (white lead). The permissible lead content in drinking water in Poland is 0.001 mg L^−1^, and is in line with WHO recommendations. In the present study, Pb was detected in only 8% of drinking water samples, and it did not exceed 0.001 mg L^−1^. In raw wastewater, however, the levels detected did not exceed 0.056 mg L^−1^. Surface waters in Woji Creek, Rivers State, Nigeria in 2019 had confirmed mean Pb concentrations of Pb of 1.316 ± 0.620 mg L^−1^ [24], and these values substantially exceeded those of the present study. Etxabe et al. (2010) and Haider et al. (2002) [28,29] observed in Spain and Austria, respectively, that lead concentrations in drinking water were higher than those in water sampled at treatment plants; these authors concluded that the poor condition of the water supply network could have resulted in lead leaching from the pipes into the water. 

High arsenic concentrations in natural water all over the world are a significant problem, and pose risks because of the toxic properties of this element. Removing arsenic can be done through oxidation, precipitation, coagulation, membrane filtration, and adsorption [30]. The arsenic limit in drinking water set by the WHO is 10 μg L^−1^. Kelepertsis et al. (2006) reported higher arsenic concentrations (125 μg L^−1^) in drinking water in eastern Thessaly in Greece [31], while Jovanovic et al. (2011) confirmed that 63% of all water samples exceeded Serbian and European standards for arsenic content in drinking water [32]. Cavar et al. (2005) reported that the mean arsenic concentrations in drinking water from three towns in eastern Croatia were 38, 172, and 619 μg L^−1^, which posed serious health risks to approximately 3% of the Croatian population [33]. Research by Tamasi and Cini (2004) indicated that arsenic concentrations in drinking water from southern Tuscany in Italy were higher than those in treatment plants [34]; these authors concluded that the poor condition of the water supply network could have caused arsenic to leach from the pipes into the water. 

Although it has been many years since legal regulations throughout the world were tightened, including in the European Union, trace elements occur in the environment, and can still pose real risks. The present study confirmed the necessity of continuing research on the effectiveness of various water treatment methods and filtration beds, and also of considering drinking water along with the food humans consume when estimating intake sources of trace elements.

## 4. Materials and Methods

### 4.1. Study Material 

**Figure 7:**
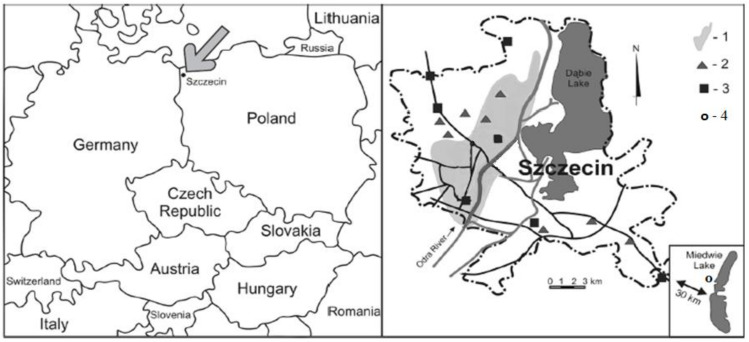
Location of the study area, 1: area supplied with water from Lake Miedwie—left-bank part of Szczecin (north, west, and downtown districts); 2: pumping stations; 3: water production plant; 4: water sampling sites at the Żelewo water production plant [36].

The materials tested in the study were raw drinking water, drinking water, raw wastewater, and treated wastewater. The raw drinking water was sampled from Lake Miedwie, which is a drinking water source and the largest freshwater reservoir in the Zachodniopomorskie (Western Pomerania) Voivodeship in Poland. The lake has an area of 35 km^2^, and is the fifth largest in Poland and the second largest in the voivodeship. The water is abstracted at two intake points in the lake at depths of 16–18 m (6 m above the lake bottom) that are located in the deep profundal zone. The intakes are fitted with 40 mm mesh gratings. Raw drinking water is treated in the Żelewo water production plant located approximately 2.5 km from Lake Miedwie (Figure 7). The wastewater tested was from the Pomorzany wastewater treatment plant, from which raw wastewater was sampled at the grating station, while treated wastewater was collected at the outflow canal. The study began in March 2017, and ran until March 2019. The parameters analyzed are presented in Table 8. 

Sampling was performed four times a month. Each time, 5 L each of raw drinking water, drinking water, raw wastewater, and treated wastewater were collected to be tested for levels of trace elements (Zn, Ni, Fe, Mn, Cu, Pb, and As), and a total of 288 samples of all types of water and wastewater were processed. Immediately after collection, water and wastewater samples were preserved with nitric acid (Merck, GmbH, Darmstadt, Germany).

#### 4.1.1. Drinking Water Treatment

Water treatment starts with the water being pumped from the lake intake point to the Żelewo water production plant through a pipeline fitted with a rotary 2 mm mesh capable of retaining particles larger than 3 mm. The water treatment process includes ozone oxidation, coagulation with PAX XL 1905 coagulant (Kemipol, Poland; with the following properties: pH—3.6 ± 0.4; alkalinity—85 ± 5%, density—1150 kg m^−3^, aluminum content—6.0 ± 0.5%; chlorides—5.0 ± 1.0%), flocculation using polyelectrolytes, and then sedimentation and filtration on a sand bed followed by an activated carbon bed (Figure 1). Finally, the water is disinfected with chlorine gas and chlorine dioxide. The treated drinking water is transported to the city of Szczecin (population of 400,000) through two 30 km mains, which supply water to 85% of the city’s inhabitants (Figure 7). 

#### 4.1.2. Treating Wastewater

The modern Pomorzany wastewater treatment plant in Szczecin has been operational for 10 years. This plant treats wastewater from approximately 50% of the urban area. The treatment stages are presented in Figure 4. The wastewater treatment process utilizes two types of coagulators: PIX 113 (Kemipol, Poland; with the following properties: total iron 11.8 ± 0.4%; density in kg m^−3^ (20 °C) 1500–1570; pH of less than 1) and PAX 16 (Kemipol, Poland; with the following properties: Al_2_O_3_ content—15.5 ± 0.4%; chlorides (Cl^−^)—19.0 ± 2.0%; alkalinity—37.0 ± 5.0%; density in kg m^−3^ (20 °C) 1330 ± 20; pH 1.0 ± 0.2). 

### 4.2. Methods 

Immediately after collection, water samples were acidified with concentrated HNO_3_ to a solution with pH < 2, with the aim of avoiding contamination and trace element precipitation. Additionally, immediately before starting the analysis, all of the samples were filtered through 0.45 μm glass fiber filter paper (Whatman, Darmstadt, Germany).

#### 4.2.1. Digestion and ICP-AES Analysis

Water for determination of the general forms of the elements selected was digested according to the PN-EN ISO 15587-2 procedure: 2005 [37]. For this, 200 mL of water was concentrated in quartz beakers on a heating plate, 3 mL of concentrated HNO_3_ (Merck, Germany) was added, and the solution was evaporated to dryness. For wastewater digestion, larger amounts of HNO_3_ (from 5 to 20 mL) were added until a light color was achieved. The resulting pellet was dissolved in 2 mL of 15% HNO_3_ and transferred quantitatively with deionized water (Barnstead Easypure UV), to a final volume of 8 mL. The nominal values of the concentrations of the trace elements analyzed were determined via inductively coupled plasma atomic emission spectrophotometry (ICP-AES; Jobin Yvon JY-24) fitted with a Meinhard TR 50-C1 nebulizer. The operational parameters of the device were as follows: generator output power 1000 W; frequency 40.68 MHz; argon as the plasma, auxiliary, and nebulizer gas at flow rates of 12.0, 1.0, and 1.1 mL min^−1^ respectively. The carrier gas flow rate was optimized to obtain maximum signal-to-background ratios. The following wavelengths were used: Zn—213.856 nm; Ni—231.604 nm; Fe—238.204 nm; Mn—257.610 nm; Cu—327.396 nm; Pb—220.353 nm; As—228.812 nm. All samples were analyzed in three analytical replications. The accuracy and precision of the method applied was determined with the certified reference material Soft Drinking Water - Metals LGC6027 (LGC Limited, Teddington, UK). The recovery of the elements tested was Zn (97.4%), Ni (98.8%), Fe (97.2%), Mn (99.0%), Cu (96.4%), Pb (98.6%), and As (97.7%). The limit of detection (LOD) and limit of quantification (LOQ) values were calculated based on the standard deviations (SDs) from 10 blank sample measurements. The LOD and LOQ values were as follows (μg L^−1^): Zn (1.15, 3.5); Ni (0.8, 2.5); Fe (0.9, 2.8); Mn (0.15, 0.45); Cu (1.1, 3.5); Pb (0.15, 0.50); As (0.6, 2.0). 

#### 4.2.2. Estimating Consumer Exposure Risk 

The risk to consumers from ingesting drinking water was estimated taking into consideration age, sex, and recommended daily water consumption [7], by determining these relationships with Equation (1), as follows:

(1) $$\mathrm{PTWI}=\mathrm{ADI}\cdot 7\;\left[µg\;\mathrm{per}\;\mathrm{kg}\;b.w.\;\mathrm{per}\;\mathrm{week}\right]$$

The parameters considered were age group, mean body weight, and daily water consumption (in liters, L) recommendations of the EFSA (2010) [7]: women (60 kg, 2 L), men (70 kg, 2.5 L), children aged 3 years (12 kg, 1.3 L), and older children (38 kg, 2 L).

#### 4.2.3. Statistical Analysis 

Statistica 13.3 was used for statistical analysis. The results are presented as medians and arithmetic means, with uncertainty demoted in standard deviations and minimum and maximum concentrations. The mean values of each parameter measured were analyzed statistically using one-way analysis of variance (ANOVA) followed by Tukey’s test (HSD post Hoc, *p* < 0.05 for determining significant differences). Correlations (*p* < 0.05) were determined among the analyzed elements and the seasons of the year in which water and wastewater were sampled. Moreover, the relationships were analyzed between the content of elements and the biochemical parameters of water and wastewater (according to the information of the Szczecin Water and Sewerage Department of 2018 and 2019), and between the different seasons of the year [35,38].

## 5. Conclusions

Trace elements in water occur as a result of natural leaching from rocks, groundwater, arable land, and industrial activities. Taking into account the amount of water consumed, particular attention is paid to the presence of toxic elements that may pose a real threat to consumers.

The research carried out in this study is important because Lake Miedwie is a reservoir of more than 90% of drinking water for the city of Szczecin, and the Pomorzany wastewater treatment plant serves most of the city’s area. 

Studies have shown that the currently used methods of water treatment and wastewater treatment do not ensure complete removal of toxic elements, but only from 28 to 97%, depending on the type of element. 

This study confirms the necessity of continuing research on the effectiveness of various water treatment methods and filtration beds, and also of considering drinking water along with the food humans consume when estimating intake sources of trace elements.

## Figures and Tables

**Table 1:** Summary statistics of trace element concentrations (mg L^−1^).

Trace Elements		Raw Drinking Water n = 288	Drinking Water n = 288	Raw Wastewater n = 288	Treated Wastewater n = 288
**Zn**	$\bar{x}$ ± SD Me (x_min_–x_max_) > LOD%	0.026 ± 0.014 0.023 (0.008–0.069) 100%	0.007 ± 0.004 0.008 (< LOD–0.021) 98%	0.218 ± 0.096 0.207 (0.088–0.536) 100%	0.080 ± 0.030 0.074 (0.035–0.192) 100%
**Ni**	$\bar{x}$ ± SD Me (x_min_–x_max_) > LOD%	0.002 ± 0.001 0.002 (< LOD–0.004) 98%	0.001 ± 0.001 0.0004 (< LOD–0.003) 52%	0.026 ± 0.012 0.023 (0.010–0.068) 100%	0.016 ± 0.006 0.015 (0.006–0.041) 100%
**Fe**	$\bar{x}$ ± SD Me (x_min_–x_max_) > LOD%	0.021 ± 0.012 0.019 (0.002–0.068) 100%	0.005 ± 0.003 0.004 (0.001–0.010) 100%	0.585 ± 0.480 0.344 (0.058–1.863) 100%	0.131 ± 0.077 0.116 (0.028–0.377) 100%
**Mn**	$\bar{x}$ ± SD Me (x_min_–x_max_) > LOD%	0.008 ± 0.004 0.007 (0.003–0.020) 100%	0.003 ± 0.002 0.003 (< LOD–0.007) 91%	0.057 ± 0.022 0.053 (0.021–0.135) 100%	0.035 ± 0.033 0.029 (0.012–0.240) 100%
**Cu**	$\bar{x}$ ± SD Me (x_min_–x_max_) > LOD%	0.004 ± 0.002 0.004 (< LOD–0.008) 98%	0.001 ± 0.001 0.001 (< LOD–0.005) 69%	0.042 ± 0.023 0.039 (0.011–0.090) 100%	0.014 ± 0.007 0.011 (0.008–0.040) 100%
**Pb**	$\bar{x}$ ± SD Me (x_min_–x_max_) > LOD%	0.003 ± 0.001 0.003 (0.0005–0.006) 100%	0.0001 ± 0.0003 0 (< LOD–0.001) 8%	0.016 ± 0.013 0.01 (< LOD–0.056) 98%	0.006 ± 0.001 0.006 (0.003–0.011) 100%
**As**	$\bar{x}$ ± SD Me (x_min_–x_max_) > LOD%	0.002 ± 0.001 0.002 (0.0001–0.004) 100%	0.001 ± 0.0005 0.001 (< LOD–0.002) 77%	0.014 ± 0.004 0.014 (0.007–0.022) 100%	0.006 ± 0.004 0.005 (0.003–0.018) 100%

Notes: all summary statistics expressed as mg L^−1^. Presented for each trace element are the mean $\bar{x}$ ± standard deviation (SD), median (Me), range (x_min_–x_max_ in brackets), and detection frequency (> LOD%).

**Table 2:** Correlations between the composition of the water tested and selected element contents (*p* < 0.05).

Parameter	Zn	Ni	Fe	Mn	Cu	Pb	As	Zn	Ni	Fe	Mn	Cu	Pb	As
	Spring							Summer						
	Drinking Water													
NH_4_^+^ (mg L^−1^)	−0.377	0.165	0.100	0.087	0.381	n.d.	−0.193	0.000	−0.210	−0.110	−0.078	−0.187	0.194	0.272
NO_3_^−^ (mg L^−1^)	0.242	−0.246	−0.135	0.121	−0.371	n.d.	0.137	0.360	0.126	−0.284	−0.329	0.169	−0.316	0.093
NO_2_^−^ (mg L^−1^)	−0.377	0.165	0.100	0.087	0.381	n.d.	−0.193	0.089	−0.037	−0.331	−0.425	−0.307	0.226	0.425
COD (mg O_2_ L^−1^)	0.119	−0.015	0.130	0.235	−0.210	n.d.	0.136	0.294	−0.401	0.179	0.057	0.323	−0.387	0.189
ClO_2_ (mg L^−1^)	−0.382	0.156	0.082	0.098	0.374	n.d.	−0.191	0.072	0.108	**0.749**	0.542	0.180	−0.158	−0.083
	Raw Drinking Water													
NH_4_^+^ (mg L^−1^)	−0.436	−0.134	−0.129	0.377	**−0.833**	−0.418	−0.375	**0.836**	0.254	0.029	−0.465	0.193	**−0.630**	−0.307
NO_3_^−^ (mg L^−1^)	0.029	0.488	0.485	−0.319	0.533	**0.666**	**0.739**	**−0.734**	−0.274	**−0.622**	−0.252	−0.186	0.452	0.308
NO_2_^−^ (mg L^−1^)	−0.162	−0.558	−0.459	0.300	−0.531	**−0.781**	**−0.640**	**0.854**	0.223	−0.063	−0.489	0.088	**−0.634**	−0.057
COD (mg O_2_ L^−1^)	−0.394	−0.252	−0.286	0.407	−0.401	**−0.622**	−0.498	0.056	0.084	0.547	0.528	0.040	−0.279	**−0.578**
	Treated Wastewater													
COD (mg O_2_ L^−1^)	0.073	−0.476	−0.086	−0.012	−0.378	0.283	0.363	**0.918**	−0.452	−0.392	0.168	−0.427	−0.208	−0.507
BOD_5_ (mg L^−1^)	**0.720**	0.261	−0.155	0.059	0.291	−0.237	−0.033	0.510	0.011	0.331	0.541	−0.309	−0.292	−0.528
P,total (mg L^−1^)	−0.046	0.492	0.081	0.014	0.394	−0.296	−0.368	0.463	−0.241	−0.109	−0.101	−0.237	−0.199	−0.547
N,total (mg L^−1^)	**−0.738**	−0.417	0.138	−0.066	−0.419	0.334	0.143	0.133	−0.078	0.120	−0.155	−0.081	−0.186	−0.425
	Raw Wastewater													
COD (mg O_2_ L^−1^)	−0.363	−0.027	−0.192	0.072	−0.435	−0.236	−0.139	0.113	−0.080	−0.385	−0.452	−0.001	0.173	−0.406
BOD_5_ (mg L^−1^)	−0.319	−0.300	0.015	0.239	−0.246	−0.244	−0.070	0.050	0.068	−0.310	−0.392	0.016	0.254	−0.302
P,total (mg L^−1^)	−0.331	0.174	−0.307	−0.060	−0.494	−0.188	−0.165	−0.009	0.077	**−0.661**	**−0.650**	−0.249	0.304	−0.240
N,total (mg L^−1^)	−0.178	0.440	−0.395	−0.252	−0.436	−0.054	−0.155	−0.100	0.239	**−0.670**	**−0.637**	−0.313	0.394	−0.078
**Autumn**							**Winter**							
	Drinking Water													
NH_4_ ^+^ (mg L^−1^)	−0.108	−0.015	−0.546	0.073	**0.714**	−0.213	−0.158	−0.544	0.158	−0.211	0.234	0.076	−0.135	0.058
NO_3_^−^ (mg L^−1^)	−0.161	0.238	−0.528	0.197	**0.611**	−0.254	−0.332	−0.494	0.060	0.012	0.017	0.033	−0.484	−0.245
NO_2_^−^ (mg L^−1^)	n.d.	n.d.	n.d.	n.d.	n.d.	n.d.	n.d.	0.038	0.125	**0.593**	**0.626**	−0.070	−0.426	−0.368
COD (mg O_2_ L^−1^)	−0.096	−0.457	−0.069	−0.274	−0.127	−0.161	−0.030	−0.089	−0.263	0.005	0.024	−0.195	0.056	−0.024
ClO_2_ (mg L^−1^)	0.352	0.157	0.328	0.205	0.115	0.302	0.310	−0.038	−0.125	**−0.593**	**−0.626**	0.070	0.426	0.368
	Raw Drinking Water													
NH_4_^+^ (mg L^−1^)	**0.576**	−0.234	0.109	**−0.686**	0.000	0.117	0.405	0.008	0.064	0.143	−0.057	**0.599**	−0.176	0.000
NO_3_^−^ (mg L^−1^)	−0.375	0.189	−0.375	−0.095	−0.110	−0.226	0.015	0.103	0.454	0.218	**−0.609**	−0.419	−0.287	−0.346
NO_2_^−^ (mg L^−1^)	0.314	0.097	0.234	−0.025	0.366	0.500	0.108	0.393	0.483	0.190	**0.820**	−0.068	0.325	0.170
COD (mg O_2_ L^−1^)	**0.576**	−0.234	0.109	**−0.686**	0.000	0.117	0.405	0.075	−0.403	0.145	−0.067	0.387	0.300	0.204
	Treated Wastewater													
COD (mg O_2_ L^−1^)	**−0.738**	**0.639**	−0.515	−0.226	0.067	0.345	0.288	−0.241	**0.756**	**0.610**	0.025	−0.486	0.566	0.445
BOD_5_ (mg L^−1^)	0.047	−0.245	0.107	**−0.621**	0.293	0.290	0.053	0.072	−0.401	**−0.706**	0.463	−0.055	**−0.686**	−0.147
P,total (mg L^−1^)	**−0.721**	0.278	−0.485	**−0.675**	0.131	0.301	0.137	0.504	0.068	0.538	−0.103	0.547	0.118	0.110
N,total (mg L^−1^)	−0.046	**0.744**	0.080	0.078	0.469	**0.708**	0.479	−0.149	**−0.693**	−0.409	−0.421	0.277	−0.119	**−0.603**
	Raw Wastewater													
COD (mg O_2_ L^−1^)	−0.328	−0.316	0.063	0.398	0.303	0.131	−0.348	0.272	−0.406	−0.056	−0.163	0.378	0.088	**−0.637**
BOD_5_ (mg L^−1^)	−0.367	−0.136	−0.094	0.417	0.203	−0.043	−0.197	0.006	−0.405	**−0.579**	**−0.579**	0.363	−0.421	**−0.766**
P,total (mg L^−1^)	0.084	−0.201	0.367	0.508	0.359	0.349	−0.183	0.355	−0.159	−0.027	−0.057	0.276	0.010	−0.182
N,total (mg L^−1^)	−0.103	−0.452	0.344	0.380	0.469	0.380	−0.449	0.265	0.031	−0.035	−0.004	0.165	−0.102	0.140

Notes: COD— chemical oxygen demand; BOD_5_— Biochemical oxygen demand; N—total nitrogen; P—total phosphorus; NO_3_^−^ nitrates (V); NO_2_^−^ nitrites (III); **Bold**—statistically significant (*p* < 0.05) correlation.

**Table 3:** Significant differences (*p* < 0.05) in the contents of elements in the water tested in different seasons of the year.

Trace Element	*p*-Value						Trace Element	*p*-Value					
	Spring	Spring	Spring	Summer	Summer	Autumn		Spring	Spring	Spring	Summer	Summer	Autumn
	vs.	vs.	vs.	vs.	vs.	vs.		vs.	vs.	vs.	vs.	vs.	vs.
	Summer	Autumn	Winter	Autumn	Winter	Winter		Summer	Autumn	Winter	Autumn	Winter	Winter
Drinking Water							Treated Wastewater						
Zn	0.236	0.87	0.833	0.659	**0.040**	0.384	Zn	0.998	0.413	0.397	0.52	0.503	1
Ni	0.357	**0.024**	0.085	0.56	0.865	0.95	Ni	0.719	0.828	0.687	0.239	1	0.218
Fe	0.779	0.915	0.948	0.921	0.935	0.927	Fe	0.658	0.223	0.972	**0.017**	0.888	0.096
Mn	1	0.969	0.918	0.978	0.935	0.998	Mn	0.995	0.985	0.695	0.937	0.549	0.878
Cu	0.736	0.371	0.109	0.929	0.571	0.902	Cu	0.832	**0**	0.902	**0.001**	0.999	**0.001**
Pb	0.477	0.887	0.887	0.887	0.887	0.854	Pb	0.76	0.928	0.577	0.982	0.99	0.906
As	1	1	0.676	1	0.744	0.725	As	**0**	0.837	0.898	**0.001**	**0.001**	0.999
Raw Drinking Water							Raw Wastewater						
Zn	0.871	0.851	0.972	0.407	0.632	0.982	Zn	**0.021**	0.857	0.207	**0.002**	**0**	0.631
Ni	0.05	0.353	0.944	0.75	**0.012**	0.133	Ni	0.93	0.153	0.884	**0.041**	0.546	0.493
Fe	0.775	0.812	0.988	1	0.922	0.943	Fe	1	0.666	0.927	0.711	0.949	0.952
Mn	**0.027**	0.895	0.078	0.133	0.968	0.301	Mn	0.959	0.276	0.534	0.553	0.828	0.965
Cu	0.854	1	0.969	0.887	0.598	0.952	Cu	0.078	0.288	0.726	0.905	**0.006**	**0.033**
Pb	0.938	0.131	1	**0.036**	0.942	0.127	Pb	0.848	**0.034**	0.545	0.194	0.953	0.446
As	0.867	1	0.807	0.885	0.352	0.784	As	**0.032**	0.225	**0.002**	0.797	0.733	0.225

Notes: **Bold**—significant differences (*p* < 0.05).

**Table 4:** Correlations among different elements in different seasons of the year.

					Spring					Summer					Autumn					Winter				
	Zn	Ni	Fe	Mn	Cu	Pb	Zn	Ni	Fe	Mn	Cu	Pb	Zn	Ni	Fe	Mn	Cu	Pb	Zn	Ni	Fe	Mn	Cu	Pb
Raw Wastewater																								
Ni	−0.248						−0.104						0.132						−0.111					
Fe	−0.147	−0.471					0.004	−0.028					0.571	−0.363					0.111	0.157				
Mn	0.163	−0.567	0.484				0.156	0.157	**0.811**				0.093	0.218	0.510				0.456	0.528	**0.769**			
Cu	0.072	−0.409	0.328	0.265			0.555	0.045	0.485	0.438			0.050	**−0.666**	0.390	0.064			0.340	−0.406	0.231	0.050		
Pb	−0.435	0.313	0.355	0.252	0.116		−0.229	0.017	−0.307	−0.256	0.007		0.535	−0.399	0.568	−0.045	0.054		−0.196	0.047	**0.666**	0.389	−0.031	
As	0.056	−0.315	0.048	0.197	**0.781**	−0.113	−0.526	0.292	0.036	0.145	−0.186	0.487	0.385	**0.815**	−0.199	0.258	**−0.606**	−0.106	0.043	0.330	0.494	0.489	0.083	0.414
Treated Wastewater																								
Ni	0.343						**−0.647**						−0.284						−0.064					
Fe	−0.449	0.183					−0.512	0.453					0.566	−0.032					0.003	0.347				
Mn	−0.118	−0.324	−0.283				0.156	0.263	0.449				0.264	0.108	0.509				−0.279	0.227	−0.233			
Cu	0.218	0.034	−0.424	**0.645**			−0.335	0.205	0.116	−0.051			0.132	0.411	**0.744**	0.231			0.316	−0.521	0.228	−0.332		
Pb	−0.254	−0.034	−0.094	0.311	−0.210		−0.246	0.521	−0.278	−0.305	0.383		−0.048	0.464	0.031	−0.370	0.258		0.018	0.344	**0.665**	−0.154	−0.130	
As	−0.156	−0.108	**0.598**	−0.211	−0.387	0.243	−0.292	−0.421	0.169	−0.162	0.049	−0.415	−0.170	0.073	−0.209	0.004	−0.114	0.558	−0.104	0.400	0.385	0.545	−0.184	0.404
Raw Water																								
Ni	−0.052						0.160						−0.137						0.305					
Fe	−0.167	0.648					0.315	0.101					0.274	0.032					**0.593**	0.112				
Mn	**−0.782**	−0.121	0.248				−0.206	−0.016	0.508				−0.097	−0.069	0.374				0.313	0.210	0.070			
Cu	0.331	0.382	0.203	−0.256			0.247	**0.633**	0.343	0.184			0.111	−0.192	**0.581**	0.273			0.063	0.131	−0.004	0.231		
Pb	0.005	0.533	0.336	−0.111			−0.562	0.205	−0.169	0.124	0.141		−0.094	**0.641**	0.093	−0.349	−0.064		−0.025	−0.310	−0.192	0.230	−0.313	
As	−0.145	**0.695**	**0.673**	−0.002	0.566	**0.774**	0.015	−0.244	−0.162	0.002	−0.177	0.088	0.356	0.244	−0.154	−0.268	−0.301	0.070	0.042	−0.263	0.091	0.207	0.490	0.143
Drinking Water																								
Ni	−0.018						−0.458						0.067						−0.106					
Fe	−0.117	0.433					0.159	0.124					−0.016	0.377					0.424	−0.097				
Mn	−0.492	0.060	0.207				−0.095	0.074	0.160				0.064	0.022	−0.418				−0.198	0.371	0.458			
Cu	−0.106	0.235	0.437	0.467			0.208	0.034	0.086	0.246			−0.148	0.025	−0.278	0.175			0.234	−0.191	0.013	−0.453		
Pb	0.016	0.141	0.288	0.241	0.153		**−0.866**	0.548	−0.301	0.090	−0.033		0.036	0.516	0.272	0.031	0.170		0.090	−0.213	−0.386	−0.326	0.465	
As	0.125	0.038	0.015	0.185	0.213	0.108	0.554	−0.561	0.066	−0.266	−0.457	−0.550	0.111	−0.091	0.387	−0.259	−0.331	0.214	0.145	−0.460	−0.256	−0.307	0.221	0.039

Notes: **Bold**— the strong correlations (*p* < 0.05).

**Table 5:** Estimated health risks associated with drinking water consumption.

Trace Element	ADI (mg kg^−1^)	PTWI (µg per kg per Week)	PTWI_1_	PTWI_2_	PTWI_3_	PTWI_4_	Mean Concentration (µg L^−1^ of Drinking Water)	PTWI_1_ (%)	PTWI_2_ (%)	PTWI_3_ (%)	PTWI_4_ (%)
As	0.0021	15	900	1050	180	570	1	1.56	1.67	5.06	2.46
Pb	0.0036	25	1500	1750	300	950	0.1	0.09	0.10	0.30	0.15
Ni	0.005	35	2100	2450	420	1330	1	0.67	0.71	2.17	1.05
Zn	0.1	700	42,000	49,000	8400	26,600	7	0.23	0.25	0.76	0.37
Mn	0.14	980	58,800	68,600	11,760	37,240	3	0.07	0.08	0.23	0.11
Cu	0.5	3500	210,000	245,000	42,000	133,000	1	0.01	0.01	0.02	0.01
Fe	0.8	5600	336,000	392,000	67,200	212,800	5	0.02	0.02	0.07	0.03

Notes: ADI—adequate daily intake [9]; PTWI—provisional tolerable weekly intake; PTWI_1_—women of 60 kg average body weight and 2 L of water consumption; PTWI_2_—men of 70 kg average body weight and 2.5 L of water consumption; PTWI_3_—children aged 3 years of 12 kg average body weight and 1.3 L of water consumption; PTWI_4_—children aged 12 years of 38 kg average body weight and 2 L water consumption.

**Table 6:** Effects of water treatment on the quantities of elements ingested in reference to nutrient reference values (NRVs).

Trace Element	$\bar{x}$ RW (mg)	$\bar{x}$ DW (mg)	NRV DW (mg)	NRV RW (%)	NRV DW (%)	Difference in NRV (%)	Difference in NRV (%)
Zn	0.026	0.007	8 (W) 11 (M)	0.32 0.23	0.09 0.06	0.23 0.17	71.88 73.91
Ni	0.002	0.001	-	-	-	-	
Fe	0.021	0.005	10 (M) 18 (W)	0.21 0.11	0.05 0.03	0.16 0.08	76.19 72.72
Mn	0.008	0.003	1.8 (W) 2.3 (M)	0.44 0.34	0.16 0.13	0.28 0.21	63.63 61.76
Cu	0.004	0.001	0.9 (W,M)	0.44	0.11	0.33	75.0
Pb	0.003	0.0001	-	-	-	-	
As	0.002	0.001	-	-	-	-	

Notes: W—women; M—men; NRV—nutrient reference value [16]; RW—raw drinking water; DW—treated drinking water.

**Table 7:** Maximum acceptable concentrations (MACs) of the trace elements tested in drinking water and wastewater.

Trace Element	MAC—Raw Drinking Water ^a^	MAC—Drinking Water ^b^	MAC—Treated Wastewater ^c^
	Content, mg L^−1^		
As	0.05	0.01	0.1
Cu	0.05	2	0.5
Ni	0.05	0.02	0.5
Pb	0.05	0.005	0.5
Mn	0.05	0.05	-
Fe	0.3	0.2	10
Zn	3	-	2

^a^—J. L. 2019 item 1747 [17], ^b^—J. L. 2017 item 2294 [6], ^c^—J. L. 2019 item 1311 [10].

**Table 8:** Parameters of the water tested [35].

Parameter	Spring		Summer		Autumn		Winter	
	$\bar{x}$	SD	$\bar{x}$	SD	$\bar{x}$	SD	$\bar{x}$	SD
Drinking Water								
pH	6.80	0.20	7.50	0.06	7.55	0.10	7.77	0.05
Alkalinity (mmol L^−1^)	3.052	0.442	3.383	0.072	3.100	0.135	3.017	0.039
NH_4_^+^ (mg L^−1^)	0.388	0.814	0.027	0.010	0.027	0.010	0.023	0.008
NO_3_^−^ (mg L^−1^)	2.963	0.405	3.487	0.172	2.360	0.744	1.898	0.051
NO_2_^−^ (mg L^−1^)	0.359	0.837	n.d.	n.d.	n.d.	n.d.	0.001	0.000
COD (mg O_2_ L^−1^)	2.73	0.29	2.63	0.10	2.67	0.13	2.48	0.09
Raw Drinking Water								
pH	8.22	0.04	7.75	0.13	7.78	0.19	8.13	0.08
NH_4_^+^ (mg L^−1^)	0.097	0.014	0.093	0.023	0.093	0.010	0.090	0.010
NO_3_^−^ (mg L^−1^)	2.570	0.216	3.027	0.277	2.145	0.930	1.692	0.070
NO_2_^−^ (mg L^−1^)	0.010	0.004	0.018	0.021	0.009	0.002	0.005	0.002
COD (mg O_2_ L^−1^)	6.98	0.13	6.82	0.14	6.67	0.05	6.57	0.10
Treated Wastewater								
pH	7.70	0.06	7.76	0.10	7.69	0.14	7.66	0.10
Alkalinity (mmol L^−1^)	4.967	2.403	2.467	1.822	4.683	2.805	4.905	1.716
NO_3_^−^ (mg L^−1^)	0.762	0.137	0.587	0.200	0.659	0.066	0.599	0.087
NO_2_^−^ (mg L^−1^)	7.53	0.36	6.74	1.32	8.20	1.08	8.67	0.99
COD (mg O_2_ L^−1^)	34.50	1.17	27.56	3.15	27.14	1.58	26.28	1.88
BOD_5_ (mg O_2_ L^−1^)	3.58	0.60	2.95	0.75	4.95	1.56	8.01	2.51
Raw Wastewater								
pH	7.92	0.04	7.82	0.21	7.81	0.12	8.00	0.11
Alkalinity (mmol L^−1^)	310.89	17.34	294.78	107.07	397.22	34.40	409.67	64.98
NO_3_^−^ (mg L^−1^)	10.11	0.97	8.46	2.55	9.49	1.02	8.89	0.85
NO_2_^−^ (mg L^−1^)	82.09	0.61	73.53	21.04	85.13	9.79	76.23	9.46
COD (mg O_2_ L^−1^)	885.6	60.48	778.1	185.02	909.4	106.71	924.1	94.77
BOD_5_ (mg O_2_ L^−1^)	437.50	64.40	385.14	132.04	425.14	96.22	378.61	95.06

Notes: $\bar{x}$—mean; SD—standard deviation; pH—pH value; COD—chemical oxygen demand; BOD—biochemical oxygen demand; NO_3_^−^—nitrates (V); NO_2_^−^—nitrites (III); n.d.—not detected.

## Data Availability

Data sharing is not applicable to this article, as no datasets were generated or analyzed during the current study that are not discussed and presented in the article.
